# Protocol for a randomized control trial of the Building Regulation in Dual Generations Program (BRIDGE): preventing the intergenerational transmission of mental illness in at-risk preschool children

**DOI:** 10.1186/s13063-023-07591-8

**Published:** 2023-09-19

**Authors:** Lara Penner-Goeke, Madeline Belows, Amanda Kristjanson, Brendan F. Andrade, Emily E. Cameron, Ryan Giuliano, Laurence Y. Katz, Lauren E. Kelly, Nicole Letourneau, Natalie Mota, Kristin Reynolds, Maureen Zalewski, Ashley Pharazyn, Leslie E. Roos

**Affiliations:** 1 University of Manitoba, Winnipeg, Canada; 2 McCain Centre for Child Youth and Family Mental Health, Centre for Addiction and Mental Health, Department of Psychiatry, University of Toronto, Toronto, Canada; 3 Depts of Pharmacology and Therapeutics, Community Health Sciences, University of Manitoba, Children’s Hospital Research Institute of Manitoba, Winnipeg, Canada; 4 University of Calgary, Calgary, Canada; 5 University of Oregon, Eugene, USA; 6 University of Manitoba, Children’s Hospital Research Institute of Manitoba, Winnipeg, Canada

**Keywords:** Maternal depression, Child mental illness, Dialectical behavior therapy, Parent skills training, Randomized controlled trial

## Abstract

**Background:**

Since the onset of the COVID-19 pandemic, the worldwide prevalence of maternal depression has risen sharply; it is now estimated that one quarter of mothers experience clinically significant depression symptoms. Exposure to maternal depression during early childhood increases the risk for the development of childhood mental illness (MI) in offspring, with altered parenting practices mediating the association between maternal depression and child outcomes. Dual-generation interventions, which aim to simultaneously treat parent and child mental health, show promise for improving outcomes for mothers with depression and their young children. The Building Regulation in Dual Generations (BRIDGE) program combines Dialectical Behavior Therapy (DBT) and parenting skills training to concurrently treat maternal depression and improve parenting practices. In pilot within-group studies, BRIDGE has led to large reductions in maternal depression and child MI symptoms. The aim of the current study is to evaluate the efficacy of BRIDGE in reducing maternal depression and child MI symptoms (primary outcomes) as well as parenting stress and harsh parenting (secondary outcomes).

**Methods:**

A three-armed randomized control trial with equal group sizes will be conducted to compare the efficacy of (1) BRIDGE (DBT + parenting skills), (2) DBT skills training, and (3) services-as-usual. Participants (*n* = 180) will be mothers of 3- to 5-year-old children who report elevated depression symptoms. Those randomized to BRIDGE or DBT skills training will complete a 16-week group therapy intervention. Assessments will be administered at pre-intervention(T1) post-intervention (T2), and 6-month follow-up (T3).

**Discussion:**

Dual-generation programs offer an innovative approach to prevent the intergenerational transmission of mental illness. The current study will add to the evidence base for BRIDGE by comparing it to a stand-alone mental health intervention and a services-as-usual group. These comparisons will provide valuable information on the relative efficacy of including parenting support in a mental health intervention for parents. The results will contribute to our understanding of how maternal depression affects children’s development and how intervening at both a mental health and parenting level may affect child and family outcomes.

**Trial registration:**

Name of registry: Clinical Trials Protocol Registration and Results System; trial registration number: NCT05959538; date of registry: July 24, 2023; available: https://classic.clinicaltrials.gov/ct2/show/NCT05959538

## Administrative information

Note: The numbers in curly brackets in this protocol refer to SPIRIT checklist item numbers. The order of the items has been modified to group similar items (see)

**Table:** 

Title {1}	Protocol for a randomized control trial of the Building Regulation in Dual Generations Program (BRIDGE); Preventing the intergenerational transmission of mental illness in at-risk preschool children
Trial registration {2a and 2b}.	Clinical Trials Protocol Registration and Results System; NCT05959538]
Protocol version {3}	Issue date: May 11, 2023
Funding {4}	This work was supported by funding from the Canadian Institute for Health Research.
Author details {5a}	Lara Penner-Goeke, University of Manitoba Madeline Belows, University of Manitoba Amanda Kristjanson, University of Manitoba Brendan F. Andrade, McCain Centre for Child Youth and Family Mental Health, Centre for Addiction and Mental Health, Department of Psychiatry, University of Toronto, Emily E. Cameron, University of Manitoba, Ryan Giuliano, University of Manitoba, Laurence Y. Katz, University of Manitoba Lauren E Kelly, Depts of Pharmacology and Therapeutics, Community Health Sciences, University of Manitoba, Children’s Hospital Research Institute of Manitoba Nicole Letourneau, University of Calgary Natalie Mota, University of Manitoba Kristin Reynolds, University of Manitoba Maureen Zalewski, University of Oregon Ashley Pharazyn, University of Manitoba Leslie E. Roos, University of Manitoba, Children’s Hospital Research Institute of Manitoba
Name and contact information for the trial sponsor {5b}	Leslie Roos Department of Psychology, University of Manitoba University of Manitoba, 66 Chancellors Cir, Winnipeg, MB, R3T 2N2. E-mail: leslie.roos@umanitoba.ca. Please contact Dr. Leslie Roos for public and scientific queries.
Role of sponsor {5c}	This funding source had no role in the design of this study and will not have any role during its execution, analyses, interpretation of the data, or decision to submit results.

## Introduction

### Background and rationale {6a}

Diagnosed in 10–15% of children worldwide, childhood mental illness [MI] remains a prominent public health concern [1]. Childhood mental illnesses, including disruptive behavior disorders, attention-deficit/hyperactivity disorder, anxiety, and mood disorders, predict low quality of life akin to that of children with chronic physical health conditions [2]. Early exposure to maternal depression is a notable risk factor for the development of childhood MI [3, 4]. Maternal depression is most common in the first few years following childbirth [5, 6]. Unfortunately, rates of clinically significant depressive symptoms among mothers doubled during the COVID-19 pandemic [7]. Clinically significant depressive symptoms are now estimated to affect 26.9% of mothers worldwide [7]. This increase suggests a current and critical need for interventions that concurrently address maternal depression and the prevention of childhood MI.

Rather than increasing risk for the development of any particular MI disorder or category, the intergenerational transmission of MI from mother to child is transdiagnostic and extends the risk broadly across many child psychopathology symptoms [4]. Inherited genetic risk, innate dysfunctional neuroregulatory mechanisms, exposure to negative maternal cognitions, behaviors, and affect, and a more stressful childhood life context are theorized to contribute to the increased risk of psychopathology in offspring [8]. Negative maternal cognition, behavior, and affect manifest in disrupted parenting practices among mothers with depression, including lower warmth and increased harshness [9, 10]. Mothers with depression tend to engage in lower-quality interactions with their young children, characterized by decreased engagement and sensitivity, more irritability, and coercive or harsh discipline [11]. Lower-quality parent–child interactions mediate the association between maternal depression and various deleterious childhood outcomes, including insecure attachment, increased risk of disruptive behavior disorders, and decreased executive functioning [12–14]. Mothers facing additional stressors, such as living in poverty, may be at particular risk for persistent depression during their child’s life and may be more likely to exhibit negative affect while parenting (e.g., hostility, anger) [11, 15]. Maternal depression is estimated to affect a quarter of mothers of young children globally, has deleterious effects on parenting practices, and increases the risk of child MI.

The COVID-19 pandemic led to both increased maternal depressive symptoms [7] and an increase in child MI, including escalations in anxiety, depression, irritability, inattention, hyperactivity, and obsession/compulsion symptoms among Canadian children [16]. Along with the threat of catching the virus itself, social and public health restrictions designed to mitigate transmission resulted in physical isolation from support and social networks, along with economic uncertainty and additional childcare responsibilities for parents. During the COVID-19 pandemic, families, and particularly mothers, were vulnerable to experiencing heightened parenting stress due to a sudden lack of family support and altered family relationships [17]. Rates of child abuse and neglect also increased during the first year of the pandemic and parents with higher depressive symptoms were at greater risk of psychologically maltreating their children [18, 19]. Restrictions related to COVID-19 have eased in many parts of the world; yet, it is unclear whether rates of maternal and child MI remain elevated. However, based on the established literature [20], it is likely that increases in maternal depression during the pandemic will have cascading effects on the development of childhood MI.

There is an acute need to address maternal and child MI. Effective intervention strategies are necessary to simultaneously treat MI in mothers while preventing the development of MI in at-risk children [21]. Several efficacious psychological treatments are available for treating depression in adults, including cognitive behavioral therapy and dialectical behavior therapy administered via group psychotherapy [22, 23]. Group-based parenting programs teach parents skills to effectively manage difficult child behaviors and have been found to improve both parenting skills and child behavior problems [24]. However, there are currently limited programs available that support maternal mental health while concurrently aiming to improve parenting, called dual-generation interventions [3, 25]. Few programs have integrated interventions for both mothers and children, creating barriers (e.g., learning multiple therapeutic techniques) for mothers seeking support in both areas [26]. Skills and knowledge acquired in programs targeting maternal MI are often non-transferable to parenting contexts, and vice versa [27]. Emerging evidence has found that programs that simultaneously target maternal MI and parenting skills are up to 50% more effective than programs that target only one aspect [25, 27]. These findings demonstrate the long-term value of dual-generation interventions to magnify the positive effects of parental interventions on child mental health outcomes [3, 26–28].

In addition to increased efficacy, dual-generation programs may decrease barriers to participation for mothers of young children. Mothers attempting to access mental health and parenting interventions cite significant obstacles for engagement, such as requiring childcare, transportation, and time off work to attend programs [29–31]. Due to barriers such as these, maternal depression is widely undertreated; in Canada, only one third of mothers with depression or anxiety report receiving treatment [32]. By simultaneously treating depression and providing parenting support, barriers to participation in the intervention may be halved (e.g., requiring childcare once a week instead of twice).

To simultaneously address maternal and child MI, the *Building Regulation in Dual Generations* (BRIDGE) group-based intervention was created. BRIDGE aims to increase intergenerational emotional regulation through pairing Dialectical Behavior Therapy (DBT) skills training with a theoretically aligned parenting skills program. DBT has been shown to be a propitious transdiagnostic treatment for underlying mechanisms of psychopathology, including emotion regulation difficulties common in depression, anxiety, and traumatic stress [33, 34]. The integration of DBT with parenting programs is a promising approach for addressing intergenerational needs. Developmentally supportive parenting is facilitated by mothers own ability to effectively regulate emotions; in so doing, a mother can simultaneously manage her own internal emotional experience and limit over-reactivity towards their child, as well as teach their child about emotions [26]. The aligned parenting content also includes best-practice behavior management training techniques, such as creating positive family routines and using positive reinforcement, framed within the context of DBT skills. BRIDGE holds promise to improve both maternal and child MI.

Two pilot studies have been conducted to evaluate BRIDGE [35, 36]. In a pre-post feasibility study, mothers with depression (*n* = 28) completed an in-person, 16-week trial of BRIDGE [36]. The intervention demonstrated good feasibility, with high retention (86% retention) and significant reductions in maternal depression (*d* = 1.02) and child MI (*d* = 1.08). In focus groups conducted at post-test, participants indicated that they were generally satisfied with the program [36]. Mothers expressed that they found the parenting skills complementary to the DBT skills, with one participant stating, “Because I understood the DBT language… it made it easier for me to understand it when we did it with children and the way it applies is just amazing.” In compliance with public health recommendations to reduce in-person contact and physical distance during the COVID-19 pandemic, a second pilot study was run to evaluate the efficacy of BRIDGE delivered via telehealth using asynchronous psychoeducational videos alongside synchronous weekly group sessions [35]. Participants (*n* = 39) showed significant reductions in child MI symptoms (*d* = 0.41), maternal depression (*d* = 1.13), and parenting stress *(d* = 0.39) from pre- to post-intervention [35]. Retention was high, with 92.3% of mothers completing the program [35]. Given these favorable results, further research comparing BRIDGE to other available programs is necessary to expand services to mothers in need.

### Objectives {7}

The current study will expand on previous evaluations of BRIDGE by conducting a randomized controlled trial (RCT) comparing (1) BRIDGE (DBT skills training + Parenting Skills), (2) DBT (DBT skills training only), and (3) services as usual (SAU). Our primary aim is to examine the effects of BRIDGE on maternal depression and child MI symptoms. We hypothesize that participants who receive the BRIDGE and DBT interventions will report fewer depressive symptoms than participants in the SAU group. Participants who receive the BRIDGE intervention are hypothesized to report fewer child MI symptoms than those in the DBT and SAU groups. Our secondary aim is to evaluate the efficacy of BRIDGE in reducing parenting stress and harsh parenting. Participants who receive the BRIDGE intervention are hypothesized to show lower levels of parenting stress and harsh parenting than those in the DBT and SAU groups.

Additional aims of the RCT are to examine the effects of BRIDGE and DBT on family relationships, other service use (e.g., hospital visits, interactions with police), and maternal psychopathology symptoms. We hypothesize that mothers who receive the BRIDGE or DBT intervention will report lower psychopathology symptoms, reduced service use, and improved family relationship quality. We will also assess participants' engagement in each intervention.

Exploratory outcomes of observed maternal sensitivity and child emotion regulation will also be examined via remote Zoom assessments. We hypothesize that mothers in the BRIDGE group will show greater maternal sensitivity and that their children will demonstrate improved emotional regulation, than those in the DBT or SAU groups. Additionally, we will invite participants’ co-parents to complete questionnaires on their own mental health and parenting. Furthermore, exploratory outcomes will include physiological indices of well-being (e.g., sleep and daily activity) measured via Fitbits that mothers will wear during the program. We hypothesize that participants who receive the BRIDGE or DBT interventions will display improved sleep quality and reduced sedentary behavior. Finally, we will also invite co-parents of enrolled participants to complete questionnaires related to their own MH and family relationships. Inviting co-parents to complete questionnaires during this trial is largely exploratory and will allow us to evaluate the feasibility of including assessments of co-parents in future trials. We hypothesize that some spill-over effects of the BRIDGE and DBT interventions may occur, such that co-parents of participants in either intervention group will show fewer MH symptoms and improved family relationship quality.

### Trial design {8}

A three-armed, parallel-design RCT with repeated measures will be used to evaluate the efficacy of the 16-week telehealth BRIDGE intervention for mental health outcomes in mothers and their children aged 3–5 years old (at study enrolment) compared to a DBT-only and SAU control group. Participants will be randomly allocated, using central randomization stratified based on telehealth session availability and location, in a 1:1:1 ratio to BRIDGE, DBT, or SAU. Primary (depression and child MI), secondary (parenting stress and harsh parenting), and some exploratory outcomes (i.e., maternal psychopathology symptoms, family relationship quality, service use) outcomes will be assessed during the enrolment period (pre-test, T1), after the last week of the BRIDGE and DBT interventions (post-test, T2), and at follow-up (T3). Exploratory outcomes of maternal sensitivity and child emotional reactivity will be assessed at T1 and T2. Physiological data will be collected via Fitbits starting at T1 and continuing until T2. Co-parent’s mental health and parenting will be assessed at T1, T2, and T3.

This trial was registered with ClinicalTrials.gov (NCT05959538). The Research Ethics Board 1 at the University of Manitoba, Fort Garry campus, has reviewed and approved this study.

## Methods: participants, interventions, and outcomes

### Study setting {9}

Participants residing in Manitoba and British Columbia (BC), Canada, will be recruited online through social media advertisements, as well as through physical posters in public locations and radio advertisements. The BRIDGE treatment condition will be delivered virtually via a secure website where psychoeducational videos will be watched, as well as through videoconference telehealth sessions. The DBT-only intervention will be conducted via videoconference telehealth using Zoom for Healthcare. Questionnaires will be administered using Research Electronic Data Capture (REDCap) hosted at the University of Manitoba, a secure, web-based software platform designed to support data capture for research studies [37, 38].

### Eligibility criteria {10}

#### Inclusion and exclusion criteria

Eligible participants must be caregivers with at least one 3–5-year-old child. The terms “mother” and “maternal” will be used throughout this paper to describe all participants; however, anyone who self-identifies as a mother or female primary caregiver (e.g., grandmothers raising grandchildren, gender diverse caregivers who identify as mothers) will be eligible to participate. Mothers must be above the age of 18, residing in Manitoba or BC, Canada, with elevated symptoms of depression (Patient Health Questionnaire [39] score ≥ 10) at the pre-survey that will occur prior to randomization. Eligible participants must also self-identify as being comfortable understanding, speaking, and reading English, having internet access, being available to attend telehealth groups, and being willing to complete T1 and T2 questionnaires. Mothers who report a suicide attempt in the past year or who have engaged in self-harm that required medical attention in the past 6 months will not be eligible to participate in the study, as the BRIDGE program is not intended to address these mental health needs. These mothers will be given a list of local mental health resources in their community that may be more suited to their needs. In addition, mothers who report a diagnosis of or treatment for post-traumatic stress disorder (PTSD), alcohol use disorder, substance use disorder, or psychotic disorder in the last year will be followed up with to evaluate whether the BRIDGE program would be suitable for their needs. Our priority is being as inclusive as possible across a diversity of mental health needs, while also acknowledging that for certain clients with severe symptomology and/or crisis-related needs, a group therapy program alone might not be an ethically appropriate service model (e.g., psychosis, dissociation due to PTSD, or substance dependence interfering with the ability to engage in programming). If a clinician judges that a participant will be able to participate and engage in BRIDGE and/or DBT, they will be considered eligible.

#### Screening and enrollment

Following informed consent, participants will complete an online eligibility screener confirming that they meet eligibility criteria, as previously defined. Participants must also meet the cut-off for moderate-to-severe depression (≥ 10) using the Patient Health Questionnaire (PHQ-9) [39]. Immediately following screener completion, eligible participants will be taken to a booking page where they will be able to schedule a Zoom assessment or Zoom tech check-in. The assessment will include both mothers and their children and involves completing a variety of tasks with a research assistant over Zoom. If participants are not interested in completing the assessment, they can complete a Zoom check-in, an approximately 10-min meeting with a research assistant to ensure participants can access Zoom and the research team can answer any questions prior to randomization. After completing either the assessment or check-in, participants will be sent the pre-intervention questionnaire. Upon completion of the questionnaire, participants will be considered enrolled.

### Who will take informed consent? {26a}

Mothers will be sent an online informed consent form which will outline the study methods in detail. Willing participants will provide electronic written consent on REDCap prior to taking the eligibility screener, completing pre-program questionnaires, and being randomized. Participants will also be provided with a separate consent form to complete a virtual Zoom assessment or Zoom tech check-in. Should any questions arise regarding the informed consent process, participants can contact the study coordinators using the BRIDGE program email address.

#### Additional consent provisions for collection and use of participant data and biological specimens {26b}

Pending positive funding, a longer-term follow-up study may be carried out. If this occurs, additional consent will be sought from all participants. No biological samples will be collected.

### Interventions

#### Explanation for the choice of comparators {6b}

The SAU condition of the study is intended to account for potential changes in depressive symptoms and other outcomes over time [40]. When compared to the BRIDGE and DBT-only conditions, the SAU condition will provide insight into whether either intervention is associated with improved outcomes (e.g., reduced depressive symptoms and parenting stress). The DBT condition will provide evidence about the benefits of providing both mental health and parenting support to mothers with MI. We are interested in examining differences in child MI symptoms and parenting stress and behaviors between participants exposed to the BRIDGE versus DBT interventions.

#### Intervention description {11a}

##### BRIDGE condition

The BRIDGE program is a manualized therapy that provides participants with parenting and DBT skills video training modules through a secure website. Mothers in the BRIDGE arm will participate in 16 weeks of 20–30 min DBT and parenting skills training videos that will be delivered asynchronously via an online website requiring a participant login. The BRIDGE condition also includes weekly synchronous 1-h virtual group therapy sessions as well as DBT and parenting skills worksheets to complete weekly. Based on participant feedback from earlier iterations of BRIDGE, participants will not be asked to complete DBT Skills Diary Cards [36].

*DBT videos* developed and recorded by our research team will provide information to participants with the goal of targeting mental health symptomatology. Video content was drawn from concepts outlined in the DBT Skills Training Manual 2nd Edition [41]. Videos will provide training skills in Mindfulness, Emotion Regulation, Distress Tolerance, and Interpersonal Effectiveness domains.

*Parenting videos* will provide mothers with parenting skills education based on best practices in evidence-based positive parenting interventions (e.g., Parent Management Training, Positive Parenting) [42, 43]. To aid the transfer of DBT skills to the parenting context, the parenting skills videos will promote skills that align with the four core DBT modules (Mindfulness, Emotion Regulation, Distress Tolerance, and Interpersonal Effectiveness) to promote self-regulation in the parenting context and positive parent–child relationships.

*Weekly virtual group therap*y will provide the clinical team with the opportunity to consult with participants about their progress throughout the program. Participants can discuss video and worksheet content in these sessions with clinicians and other parents in the program during each session. The clinical team will consist of two Master’s or Ph.D. level clinical psychology trainees and a parent peer coach, a trained mother of young children who completed the BRIDGE program in the past.

*Mood tracking* will be completed using a brief weekly survey including questions on depression, parenting stress, positive mood, and recent stressful experiences. Participants will be provided with a weekly score for their depressive symptoms and parenting stress. Participants will track and graph these weekly scores in the program handbook provided to them by the research team.

##### DBT only

Mothers in the DBT arm will not receive parenting skills training and will participate in 16 weeks of DBT skills training only led by two Master’s or Ph.D. level clinical psychology trainees. Participants in the DBT Skills condition will receive training following the DBT Skills Training Manual 2nd Edition [41] through weekly, synchronous 1.5-h virtual group therapy sessions, as well as worksheets to complete weekly. Mindfulness, Emotion Regulation, Distress Tolerance, and Interpersonal Effectiveness skill domains will be covered. Participants will also be instructed to complete weekly Diary Cards to track DBT skills use each week [40].

*Mood tracking* will be completed using a brief weekly survey including questions on depression, parenting stress, positive mood, and recent stressful experiences. Participants will be provided with a weekly score for their depressive symptoms and parenting stress. Participants will track and graph these weekly scores in the program handbook provided to them by the research team.

##### SAU

Participants in the SAU condition will receive a list of local mental health and parenting resources, curated by our research team, and can access any intervention or resource they would like throughout the duration of the program.

#### Criteria for discontinuing or modifying allocated interventions {11b}

Should any participant disclose worsening symptoms (e.g., suicidal behavior) during the program, the clinical team will consider whether continued participation in the program is in the best interest of the participant. Participation in the program may also be discontinued if a participant engages in repeated violations of the terms of use regarding virtual group therapy sessions (e.g., breaking confidentiality by having other people visible in the background, leaving unexpectedly without alerting a clinical coach). Furthermore, if the clinical team notices that a participant’s clinical disposition or mental health needs change over the course of treatment, the clinical team may provide a referral to another mental health provider or clinic.

#### Strategies to improve adherence to interventions {11c}

To encourage engagement with the program, participants will be required to confirm their availability to attend weekly virtual group therapy sessions. Before starting the BRIDGE or DBT intervention, participants will attend a Zoom orientation meeting with a clinical coach. The purpose of the orientation meeting is to welcome participants to the program, explain the program components, review DBT assumptions and guidelines, assess safety and risky behaviors (e.g., suicidal or self-harm behavior, substance use), and answer questions. As some topics are not discussed in group given the potential to negatively affect other group members, the orientation meeting serves to assess the risk for drop-out, validate the importance of these safety concerns, and inform participants on how to reach out to facilitators individually on these topics (e.g., pre- or post-group check-ins, individual meetings outside of group time). As standard practice, participants in the BRIDGE and DBT conditions will be sent an email or SMS text reminder once per week for that week’s group and content. Should participants not present to their respective groups, a clinical coach will contact them at the 5-min mark to provide the Zoom link and invitation to join. Additional follow-up will occur for participants who miss the session and are non-responsive to the reminder. Contact will occur through multiple methods and with the use of validation and encouragement to support participants returning to the group. Participants will be informed that missing four sessions without being responsive to communication and efforts to return to the group will result in discontinued treatment.

To ensure adherence to the DBT Skills group protocol and assumptions during weekly therapy sessions, our team created a clinician-reported adherence measure. The measure was adapted from Harned and colleagues [44] for group therapy, with the integration of relevant resources [45, 46]. The measure was reviewed by experts in DBT (and co-authors on the current study) and refined. The resulting measure consists of 30 items rated as adherent, not adherent, or not applicable. Clinicians in both the BRIDGE and DBT groups will complete the adherence measure after each therapy session.

#### Relevant concomitant care permitted or prohibited during the trial {11d}

Participants will remain eligible for inclusion in the study if they receive concomitant care for mental health or parenting concerns; this will be documented and controlled for in the analysis as appropriate. Participants who are identified as having mental health concerns beyond depressive symptoms will be given access to a resource list of mental health and parenting supports and services which they may access during the program. Participants may continue with or begin additional clinical care (e.g., individual psychotherapy, psychiatric medications) throughout their study involvement. Additionally, participants who present with elevated suicidality or substance use either during the screening process or throughout the intervention will be offered appropriate care (e.g., monthly individual meetings with a clinician) or referred for additional clinical care.

#### Provisions for post-trial care {30}

If additional treatment is deemed necessary, a referral to another provider or clinic will be made and a list of community resources will be provided.

### Outcomes {12}

#### Primary outcomes (the specific measures utilized are described below in data collection and management)

The primary outcome of this study is mean change in depressive symptoms in mothers from pre- to post-intervention as measured by a self-report questionnaire [39]. Another primary outcome of this study is mean change in child behavioral problems from pre- to post-intervention, measured by parent-report questionnaire [47].

#### Secondary outcomes

A secondary outcome is mean change in parenting stress from pre- to post-intervention, which will be assessed using a self-report questionnaire [48]. We will also measure mean change in harsh parenting, using a self-report questionnaire [49].

#### Exploratory outcomes

Exploratory outcomes of this study include the mean change in family relationship quality from pre- to post-intervention, which will be assessed using self-report questionnaires measuring parenting qualities and strategies used by mothers, family support, and co-parent relationship quality. Additionally, mean change in maternal mental health symptoms will be assessed using self-report questionnaires measuring symptoms of various mental illnesses. Participants will also be asked to report on their recent exposure to stressful life events. Health and social service use will also be assessed at pre- and post-intervention.

In the intervention groups, we will measure participants’ engagement in program components, such as attendance to group and self-reported video watching (in the BRIDGE group).

Additional exploratory outcomes will consist of observational data of maternal sensitivity and child emotion regulation, measured by a virtual Zoom assessment with mothers and their children. Exploratory outcomes will also include co-parent mental health and parenting, and physiological indices of well-being (e.g., sleep and daily activity) collected using wearable Fitbit watches for monitoring participants’ heart rate.

### Participant timeline {13}

Week 0 (T1): Eligible consenting participants will receive a notice of enrollment via email. Exploratory outcomes will be assessed at this time point via opt-in virtual Zoom assessments. Participants will complete a 45-min baseline questionnaire, including primary, secondary, and exploratory outcomes. The T1 timepoint will be approximately 8 weeks prior to the start of the intervention.

Week 8: Participants randomized to either intervention condition will be sent an email regarding website login information. Participants will attend a clinical orientation meeting with clinical coaches.

Weeks 9–24: Participants in the BRIDGE condition will watch DBT and parenting skills training videos and complete the accompanying worksheets weekly. Participants in this condition will also complete brief weekly symptom tracking questionnaires and attend weekly virtual group therapy sessions.

Participants in the DBT condition will attend weekly virtual group therapy sessions and will complete educational worksheets. They will also complete brief weekly symptom tracking questionnaires.

Week 25 (T2): Participants will complete questionnaires assessing primary, secondary, and exploratory outcomes. Additional exploratory outcomes will be assessed via virtual Zoom assessment and co-parent questionnaire completion.

Week 25 + 6 months (follow-up): Six months post-intervention, participants will complete a 45-min follow-up questionnaire to assess prolonged changes in primary and secondary outcomes, as well as exploratory outcomes related to family relationships, maternal mental health symptoms, recent stressful experiences, and health and social services usage (Table 1).

**Table 1:**
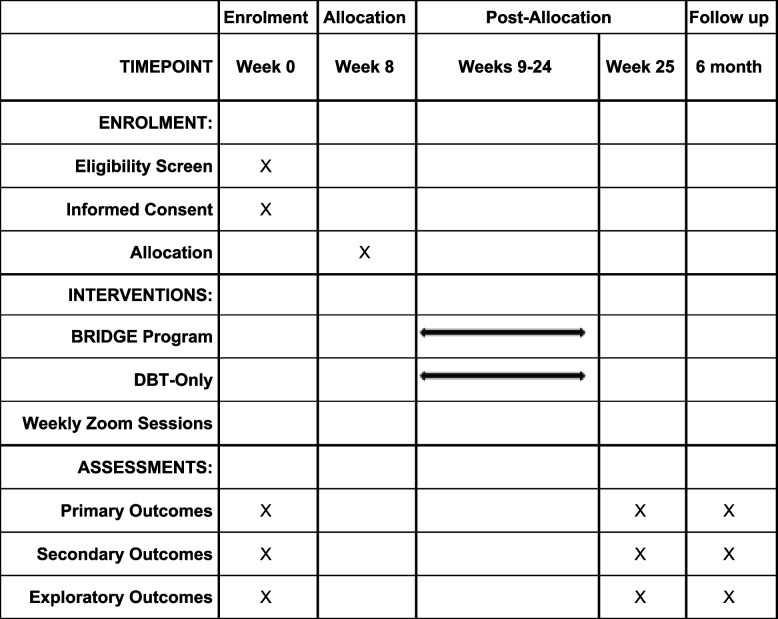
Standard Protocol Items: Recommendations for Interventional Trials (SPIRIT) schedule of enrolment, allocation, intervention, and assessments

### Sample size {14}

A sample size of 180 participants will be sufficient to assess clinically meaningful reductions in both maternal depressive and child MI symptoms between groups. Based on the feasibility pilot data, we assume an effect size of *d* = 1.02 in improvement on maternal depression symptoms for both intervention groups (BRIDGE and DBT Skills-Only) [36]. A sample size of *n* = 60 per group will provide very high power (> 99%) to compare intervention groups to SAU. Based on pilot data on child MI symptoms, we assume a within-group SD of 13 on the CBCL and a mean improvement of 14 points [36]. As we anticipate little to no improvement in the SAU arm, we assume an effect size of* d* = 1.08 between BRIDGE and SAU and an effect size of *d* = 0.54 between BRIDGE and DBT-Only (i.e., an effect size 50% smaller than for BRIDGE, based on meta-analyses on the benefit of dual generation programs) [27]. A sample size of *n* = 60 per group will provide very high power (> 99%) to compare BRIDGE with SAU and adequate power (75%) to compare BRIDGE with DBT Skills-only.

### Recruitment {15}

Recruitment will occur online through multiple sources including online advertisements posted to various social media platforms including the Hearts and Minds Facebook, Instagram, and Twitter accounts. Recruitment materials will also be provided to community agencies, healthcare facilities, and childcare centers. Individuals who meet all eligibility criteria and consent to randomization will be invited to participate. Eligible participants will receive compensation of up to $200 CAD for engaging with the study for its entire duration. This includes a $25 CAD honorarium for completing the pre-program questionnaires, $25 CAD for completing the optional pre-program Zoom assessment, $50 CAD for completing the post-program questionnaires, $50 CAD for the optional post-program Zoom assessment, $25 CAD for completing the follow-up questionnaires, and an additional $25 CAD for participants in the BRIDGE condition who complete at least 75% of the weekly surveys.

### Assignment of interventions: allocation

#### Sequence generation {16a}

Participants will be randomly allocated in a 1:1:1 ratio to the BRIDGE intervention, DBT-Skills intervention, or SAU. Stratified randomization will be applied based on (a) participants’ availability for virtual therapy timeslots (i.e., lunchtime or evening group) and (b) province of residence. Randomization will occur in blocks to ensure approximately equivalent group sizes.

#### Concealment mechanism {16b}

A statistician who is not affiliated with the BRIDGE clinical research team will create the allocation table. Next, a REDCap administrator, who is not affiliated with the BRIDGE clinical research team, will upload the randomization lists to Redcap and finalize the project. The REDCap randomization module ensures that once the REDCap project is finalized, no changes to the randomization tables can be made. Therefore, the allocation sequence will be concealed from all personnel affiliated with the BRIDGE clinical research team until interventions are assigned.

#### Implementation {16c}

Randomization will be conducted using the REDCap randomization module [37, 38]. Randomization lists will be uploaded to REDCap prior to the first randomization block and will not be changed throughout the project’s duration. Following the completion of baseline questionnaires, a BRIDGE research assistant will notify participants of their treatment condition based on the computer-generated assignment.

### Assignment of interventions: blinding

#### Who will be blinded {17a}

Given the heterogeneity in the administration of the intervention and control arms, participants and clinicians will not be blinded. The research assistants assigned to code observational measures in assessments will be blinded to participants’ treatment assignments.

#### Procedure for unblinding if needed {17b}

Intentional unblinding will not occur for research assistants coding observational measures; if a research assistant unintentionally discovers a participant's allocation while watching a video, the video will be assigned to a different research assistant.

### Data collection and management

#### Plans for assessment and collection of outcomes {18a}

##### Primary outcomes

Depressive symptoms will be measured using the PHQ-9 [39]. The PHQ-9 is a 9-item self-report questionnaire with possible scores ranging from 0 to 27, with higher scores indicating greater symptom severity. Scores ≥ 10 will be considered clinically significant. The PHQ-9 has been used extensively for depression screening and demonstrates high specificity (92–94%) for identifying depression in mothers of young children [47]. Changes in child MI symptoms will be assessed using the Child Behaviour Checklist (CBCL) [48]. The CBCL is a parent-report questionnaire that measures child functioning across internalizing and externalizing symptoms. This measure also includes a Total Problem Score (TPS) as well as externalizing and internalizing problem scales. Higher scores on each of these subscales indicate a greater degree of child behavior and emotional problems. Possible scores on the TPS scale range from 0 to 200. The CBCL has demonstrated good validity and reliability [48]. It is one of the most widely used rating scales for child psychopathology and behavior and is a useful screener for childhood psychiatric disorders [49, 50].

##### Secondary outcomes

Parenting stress will be measured using the Parenting Stress Index-Short Form (PSI-SF) [51]. The PSI-SF is a self-report questionnaire that requires respondents to answer questions regarding their overall experience with parenting stress using a 5-point Likert scale ranging from 1 (*strongly disagree*) to 5 (*strongly agree*). Possible total scores range from 36 to 180, with scores above 90 indicating clinically significant distress [51]. The PSI-SF has been demonstrated to have good validity, test–retest reliability, and high internal consistency and is useful in clinical applications with mothers of young children [51, 52]. Harsh parenting will be measured using the Overreactivity subscale of the Parenting Scale [53]. The Overreactivity subscale contains 10 items related to harsh parenting behaviors. Harsh parenting includes expressing inappropriate anger, irritability, or meanness towards one’s child. Each item contains a parenting situation (e.g., “When my child misbehaves…”) and ask parents to rate how they would respond on a 7-point scale, using bimodal anchors that represent effective (e.g., “I speak to my child calmly”) and ineffective (e.g., “I raise my voice or yell”) parental responses. The Overreactivity subscale has strong internal consistency and responses have been demonstrated to correlate with child behavior problems in diverse populations [53].

##### Exploratory outcomes

Family relationship quality will be measured using the Couples Satisfaction Index (CSI-4) [54], the Multidimensional Scale of Perceived Social Support (MSPSS) [55], and the Coping with Children’s Negative Emotions Scale (CCNES) [56]. The CSI-4 is a 4-item scale with excellent internal consistency (0.90) and convergent and divergent validity in samples of women with depression [57]. The 12 items on the MSPSS demonstrate good internal reliability (0.88) [58]. The 6 subscales that make up the CCNES have acceptable internal reliability, ranging from 0.69 to 0.85 [56].

Maternal mental health will be measured using a variety of self-report measures including the Self-Compassion Scale-Short Form (SCS-SF) [59], the Generalized Anxiety Disorder 7-Item Scale (GAD-7) [60], the Patient-Reported Outcomes Measurement Information System (PROMIS) Anger Short Form (SF) [61], the PROMIS-Sleep Disturbance Subscale SF [62], the Alcohol Use Disorder Identification Test (AUDIT) [63], the Cannabis Use Disorder Identification Test (CUDIT) [64], and the Recent Stress Experiences checklist. The SCS-SF contains 12 items and shows strong internal consistency (0.86) [59]. The GAD-7 is a 7-item scale with strong internal consistency (0.89) [65]. The PROMIS Anger SF is a 5-item scale with strong internal consistency (0.90), as well as moderate convergent validity with other measures of aggression (*r* = 0.51) [61]. The PROMIS Sleep Disturbance SD contains 8 self-report items relating to participants’ sleep quality [62]; it has demonstrated strong psychometric properties in a variety of clinical and non-clinical populations [66]. AUDIT is a 10-item questionnaire with strong internal consistency (0.85) [67]. The CUDIT consists of 8 items with an internal consistency of 0.91 [64]. The RSE was developed by authors, based on recommendations from the JBP Research Network on Toxic Stress at Harvard’s Center on the Developing Child, to measure familial exposure to various stressors which may affect participants.

An additional scale created for use in this project will assess health and social service utilization. The scale asks about the number and type of participant experiences with various health and social services (e.g., hospital visits, Child and Family Services contacts, visits to the public library) in the previous 3 months.

Program engagement will be assessed in a variety of ways. In both the BRIDGE and DBT Skills-only groups, clinicians will take attendance during therapy sessions. In the BRIDGE group, we will extract aggregate data on the number of views on the psychoeducational videos using Google Analytics. At T2, we will ask participants in the BRIDGE group to report about their use of the videos, homework assignments, and mood tracking throughout the intervention.

Exploratory outcomes will be assessed using observational measures of maternal sensitivity and child emotional regulation through virtual assessment videos. Maternal sensitivity will be coded using the Maternal Q-Sort developed by Pederson and colleagues [68]. Child emotions will be coded using a coding scheme developed by our research team. Child emotion codes were created in a systematic nature based on similar virtual assessments conducted by our team in the past. Codes include observations of children’s emotional cues (e.g., frowning, smiling) and verbal statements (e.g., “I hate this game,” “That was the best game!”) during and following an emotion-inducing task. Trained undergraduate coders will be required to reach 70% reliability on five separate videos when compared to an expert coder (Cronbach’s intra-class coefficient) [69] before coding independently. A total of 25% of all video segments coded will be double-coded by an expert coder to ensure the team maintains a reliability of 70% or higher.

An additional exploratory outcome includes physiological indices of well-being, which will be measured using Fitbit devices to monitor heart rate, sleep quality and duration, and physical exercise throughout the intervention’s duration. Finally, co-parent mental health and relationship quality will be measured via questionnaires (PHQ-9, CBCL, PSI-SF, CSI-4, MSPSS, CCNES, Parenting Scale Overreactivity, SCS-SF, GAD-7, PROMIS Anger, RSE, PROMIS Sleep, AUDIT, and CUDIT).

##### Descriptive measures

Descriptive measures to be collected include socioeconomic and demographic information including age, highest level of education (high school diploma or lower vs. post-secondary education), household income, marital status (married or in a common-law relationship vs. other), number and ages of all children, and type of residential community (e.g., urban area vs. rural area). Additionally, we will collect information from mothers about the history and severity of their depression (e.g., age of onset, length of episodes). We will also collect information about the target child’s health, including information about their birth, physical health, mental disorder diagnoses, and medication use.

#### Plans to promote participant retention and complete follow-up {18b}

Participants will receive weekly email or SMS text reminders containing information for telehealth sessions. Participants will receive an additional $10 CAD if they complete the post-intervention and follow-up surveys in a timely manner.

#### Data management {19}

REDCap is managed by The Centre for Healthcare Innovation which acts as a hired consultant on the proposed study and will facilitate secure data collection and management. RCT data will be stored on a secure server in accordance with the University of Manitoba’s PHIA policies.

#### Confidentiality {27}

In accordance with the University of Manitoba ethics guidelines, participant confidentiality will be maintained through all phases of the study. All data from the study will be accessed exclusively by research team members who are trained in the University of Manitoba ethics protocols have completed training on the Public Health Information Act, and have taken an oath of confidentiality.

Assessment data will be stored on REDCap or password-protected University of Manitoba data servers. Documents containing identifying information will be stored securely on password-protected University of Manitoba data servers and will be accessed by a limited number of research coordinators. Questionnaire, Fitbit, and assessment data will be linked to de-identified participant ID numbers.

Telehealth sessions will be hosted on the secure Zoom Healthcare platform and will be password protected. Due to the group-based nature of the interventions, participant anonymity during sessions cannot be guaranteed. During the orientation session, clinicians will outline the potential limits to confidentiality and anonymity. Participants will be asked to share only their first names, avoid sharing about other members of the group with anyone outside of the group, and attend sessions from a private location. Further, telehealth sessions will not be recorded or included in assessment, although attendance and client notes will be recorded and stored.

#### Plans for collection, laboratory evaluation, and storage of biological specimens for genetic or molecular analysis in this trial/future use {33}

Not applicable. No biological specimens will be collected.

## Statistical methods

### Statistical methods for primary and secondary outcomes {20a}

Analyses for primary and secondary outcome variables, as well as exploratory outcome variables, will compare the changes over time between (a) the BRIDGE vs SAU groups and (b) BRIDGE vs DBT-only groups, each at *a* = 0.025 (combined type I error rate to 0.05). Linear models accounting for pre-intervention scores will be used as the primary analysis method. The persistence of effects at the 6-month follow-up will be examined using time-by-intervention interactions in a linear mixed-effects model, accounting for within-subject serial correlations and between-subject effects of the intervention and covariates. Standardized effect sizes for linear mixed models will be derived based on recommendations for linear mixed models [70, 71]. Standardized effect sizes of 0.20, 0.50, and 0.80 will be used to interpret small, medium, and large effects, respectively [70]. The research team will also investigate if there is baseline moderation by symptom severity to examine whether the BRIDGE intervention is more effective than the DBT-only or SAU interventions for participants who have higher symptom levels at pre-intervention.

### Interim analyses {21b}

Interim analyses of primary and secondary outcomes will be conducted at the mid-way point of the RCT, when approximately 90 participants have completed the post-intervention questionnaires (T2). Minor changes in intervention delivery (e.g., offering optional in-person group therapy, updating DBT homework, changing video production) may be made based on results from interim analyses. Any changes to intervention delivery will be documented in a protocol deviation document.

### Methods for additional analyses (e.g., subgroup analyses) {20b}

Subgroup analyses may be conducted based on attendance at telehealth sessions to evaluate whether those who do not attend sessions regularly achieve the same benefit as those who do. Additional subgroup analyses, based on emerging research questions of interest, may be conducted as well.

### Methods in analysis to handle protocol non-adherence and any statistical methods to handle missing data {20c}

All analyses will employ intent-to-treat (ITT) methods, meaning that all participants randomized, whether they receive their allocated intervention or withdrew from the study, will be included [72]. Additionally, analyses using only data from participants who completed the intervention will be conducted for comparison purposes. In these analyses, missing data will be handled using maximum likelihood, which estimates values based on all available data and thus produces unbiased model parameters and standard errors.

### Plans to give access to the full protocol, participant level-data and statistical code {31c}

As principal investigator, Dr. Roos will be primarily responsible for data management. Data analysis will occur independently. Data will not be released to any third party (including the funder) before the trial is completed. De-identified participant data will be made publicly available after the initial publication of results on an open-access platform and will also be available upon request from the primary investigator. Statistical code will also be available upon request following the publication of the results.

### Oversight and monitoring

#### Composition of the coordinating center and trial steering committee {5d}

Dr. Roos is the primary investigator and will be responsible for the overall management of the project. Dr. Roos brings expertise in clinical trial interventions for maternal MI and parenting. Dr. Katz is a child and adolescent psychiatrist who will advise on health service integration and training in DBT Skills, for which he is a certified trainer. Dr. Zalewski is an associate professor at the University of Oregon and is a Linehan Board Certified DBT therapist. She will provide insight on maternal MI, family DBT interventions, and DBT fidelity monitoring. Dr. Mota, also a professor of clinical psychology, will consult on trauma-informed care for women in the DBT therapeutic context. Dr. Cameron, a post-doctoral fellow, provides expertise in DBT and parenting interventions. She will co-lead intervention training and provide clinical support and supervision on the DBT consult team. Expertise in early childhood parenting and maternal sensitivity measurement will be provided by Dr. Letourneau, the Alberta Children’s Hospital Foundation Chair in Parent and Child Mental Health. As well, Dr. Kelly is a clinical trialist at the Centre for Healthcare Innovation who will consult on trial design and provide expertise in best-practice trial methodologies for maternal and pediatric clinical health trials. Dr. Andrade will provide expertise on child behavioral interventions and clinical trial methodology. He is a clinician-scientist at the Centre for Addictions and Mental Health and an Associate Professor of Psychiatry at the University of Toronto. Dr. Giuliano is a developmental psychologist with expertise in cognitive neuroscience. He will advise on the collection, management, and analyses of physiological indices of well-being collected via Fitbits. Dr. Reynolds, a professor of clinical psychology, provides expertise in patient engagement, knowledge translation, and health service integration.

#### Composition of the data monitoring committee, its role and reporting structure {21a}

The current trial does not have a formal data monitoring committee. Instead, research assistants will meet weekly with the principal investigator, Dr. Roos, to review ongoing trial activities. The clinical team will meet weekly during the intervention to discuss any questions or concerns that arise during telehealth sessions.

#### Adverse event reporting and harms {22}

No adverse events were reported in the pilot studies of the BRIDGE intervention. A risk management protocol exists for mental health crises or child maltreatment concerns. If a participant in the BRIDGE or DBT interventions reports increasing psychological distress throughout the intervention, a clinician will meet with them individually and may connect them to more intensive mental health services if necessary. The primary investigator will be informed of any participant crises or concerns and respond appropriately. Any adverse events will be reported to the University of Manitoba Research Ethics Board and our research team will comply with their instructions.

#### Frequency and plans for auditing trial conduct {23}

If the Research Ethics Board requests an audit, all procedures and instructions will be followed. Currently, the study team has no plans for independent auditing of trial conduct.

#### Plans for communicating important protocol amendments to relevant parties (e.g., trial participants, ethical committees) {25}

Any proposed changes to the study protocol will be submitted as protocol amendments to the Research Ethics Board. If requested by the Research Ethics Board or relevant to mothers’ participation, we will inform enrolled participants of changes via a consent appendium delivered via email.

### Dissemination plans {31a}

Regardless of the magnitude or direction of effects, primary outcome results from this trial will be disseminated to both academic and non-academic audiences within 1 year following final data collection. Several publications presenting primary, secondary, and exploratory outcomes will be prepared and submitted to open-access, peer-reviewed journals. Prepared articles will be posted on open science platforms as pre-prints. Results will be presented at local (e.g., Manitoba Children’s Hospital Research Day), national (e.g., Canadian Psychological Association), and international (e.g., Society for Research in Child Development) conferences. For non-academic audiences, lay summaries and infographics will be created and distributed via our lab social media and existing connections with child and family service agencies (e.g., Acorn Family Place). All interested study participants will receive a summary of the results via email. We will work with our networks to distribute results in local and national media. If results show the hypothesized superiority of the BRIDGE program on maternal and child MI outcomes, we will explore future funding opportunities for scaling-up and integrating the BRIDGE program into existing health services infrastructure.

## Discussion

Dual-generation interventions that address both maternal and child MI are advantageous to (a) address common barriers to participation in a mental health intervention for mothers, and (b) transfer learned mental health skills to the parenting context, to increase maternal sensitivity and improve child MI outcomes. The BRIDGE intervention combines DBT skills with evidence-based parenting skills for mothers of preschool-aged children, in order to decrease maternal depression symptomology, child MI problems, parenting stress, and harsh parenting. The program is the first to incorporate DBT and parenting skills for parents of young children. The current RCT builds on previous pilot studies to provide additional evidence on the efficacy of BRIDGE as compared to a standalone maternal mental health intervention (e.g., DBT skills only) and SAU. BRIDGE will be delivered using an eHealth model. In our previous trials, eHealth has been identified by mothers as a preferred method of service delivery; however, the efficacy of eHealth-delivered interventions for maternal depression remains understudied. Our results will provide additional evidence on the efficacy of dual-generation and eHealth interventions for mothers of young children. Given the high prevalence and detrimental effects of maternal depression on young children [3, 7], intervening with both a mental health and parenting skills intervention is expected to yield benefits for the long-term health of children and mothers. Findings from this RCT may inform the integration of BRIDGE or similar dual-generation programs into current health services.

### Trial status

Recruitment began in October 2022 and is expected to continue until December 2023. The randomization of 78 participants occurred in December 2022 and January 2023. A first round of the BRIDGE and DBT-Only interventions was conducted from January 17 to May 2, 2023, with post-intervention assessments currently underway. A second and third round of interventions are planned for November 2023 and January 2024, respectively. All data collection is expected to be complete by October 2024*.*

## Data Availability

As principal investigator, Dr. Roos will be primarily responsible for data management. Data will not be released to any third party (including the funder) before the trial is completed. De-identified participant data will be made publicly available after the initial publication of results on an open-access platform and will also be available upon request from the primary investigator. Statistical code will also be available upon request following the publication of the results.

## Abbreviations

AUDIT: Alcohol Use Disorder Identification Test
BC: British Columbia
BRIDGE: Building Regulation in Dual Generations
CAD: Canadian dollars
CCNES: Coping with Children’s Negative Emotions Scale
COVID-19: Coronavirus disease 2019
CBCL: Child Behaviour Checklist
CSI-4: Couple Satisfaction Index - 4-item
CUDIT-R: Cannabis Use Disorder Identification Test- Revised
DBT: Dialectical Behavior Therapy
GAD-7: Generalized Anxiety Disorder – 7-item
ITT: Intent-to-treat
MI: Mental illness
MSPSS: Multidimensional Scale of Perceived Social Support
PHIA: Personal Health Information Act
PHQ-9: Patient Health Questionnaire
PI: Principal investigator
PROMIS: Patient-Reported Outcomes Measurement Information System
PSI-SF: Parenting Stress Index- Short Form
PTSD: Post traumatic stress disorder
RCT: Randomized controlled trial
REDCap: Research Electronic Data Capture
RSE: Recent stressful experiences
SAU: Services as usual
SCS-SF: Self-Compassion Scale Short Form
SMS: Short message/messaging service
SPIRIT: Standard Protocol Items: Recommendations for Interventional Trials
TPS: Total Problem Scale
